# Influenza A Virus Induces Autophagy by Its Hemagglutinin Binding to Cell Surface Heat Shock Protein 90AA1

**DOI:** 10.3389/fmicb.2020.566348

**Published:** 2020-10-07

**Authors:** Xingbo Wang, Tuyuan Zheng, Lulu Lin, Yina Zhang, Xiran Peng, Yan Yan, Jing Lei, Jiyong Zhou, Boli Hu

**Affiliations:** ^1^MOA Key Laboratory of Animal Virology, Department of Veterinary Medicine and Center of Veterinary Medical Sciences, Zhejiang University, Hangzhou, China; ^2^Institute of Immunology and College of Veterinary Medicine, Nanjing Agricultural University, Nanjing, China

**Keywords:** influenza A virus, hemagglutinin (HA), HSP90AA1, virus entry, autophagy

## Abstract

Autophagy can be utilized by the influenza A virus (IAV) to facilitate its replication. However, whether autophagy is induced at the stage of IAV entry is still unclear. Here, we report that IAV induces autophagy by hemagglutinin (HA) binding to heat shock protein 90AA1 (HSP90AA1) distributed on the cell surface. Virus overlay protein binding assay and pull-down assay indicated that IAV HA bound directly to cell surface HSP90AA1. Knockdown of HSP90AA1 weakened H1N1 infection. Incubation of IAV viral particles with recombinant HSP90AA1 or prior blockade of A549 cells with an anti-HSP90AA1 antibody could inhibit attachment of IAV. Moreover, we found that recombinant HA1 protein binding to cell surface HSP90AA1 was sufficient to induce autophagy through the AKT-MTOR pathway. Our study reveals that the HSP90AA1 on cell surface participates in IAV entry by directing binding to the HA1 subunit of IAV and subsequently induces autophagy.

## Introduction

Influenza A virus (IAV), a negative-sense single-stranded RNA virus, belongs to the Orthomyxoviridae family (Peiris et al., 2007). Influenza A virus causes annual seasonal epidemics and leads to great economic losses worldwide (Zhou et al., 2009). This virus attaches to the terminal sialic acid (SA), which has long been recognized as the primary receptor on the cell surface (Sauter et al., 1992; Fukuzawa et al., 2011), and through the viral hemagglutinin (HA) glycoprotein and initiates the entry process via multiple endocytic pathways (Hers, 1966; Skehel and Wiley, 2000). The HA gene encodes HA0, which can be cleaved into HA1 and HA2 after specific proteolytic cleavage. HA1 and HA2 then form the fusogenic homotrimer structure, which is anchored in the viral envelope. The HA1 subdomain forms the globular head of this structure and is responsible for the binding of IAV to SAs on the host cell membrane and subsequently initiates virus entry by virus-cell adsorption and endocytosis (Yang et al., 2013). Recently, a new protein nucleolin (NCL) in the A549 cells has been shown to play a role in virus internalization (Chan et al., 2016). However, whether IAV utilizes other putative receptors or certain receptor components to facilitate entry is still largely unknown.

Autophagy functions to remove intracellular damaged organelles, protein aggregates, and pathogens (Kudchodkar and Levine, 2009). However, several studies have shown that autophagy induced by IAV infection is critical for its replication (Zhou et al., 2009; Zhirnov and Klenk, 2013; Wang et al., 2019). Interestingly, IAV-encoded NS1 promotes the formation of autophagosomes by upregulating the expression of the viral proteins HA and M2 (Zhirnov and Klenk, 2013). Moreover, M2 and viral nucleoprotein (NP) enhance autophagy through the AKT/mTOR pathway (Wang et al., 2019). Autophagy can be induced by pattern recognition receptors (PRRs) sensing and detecting pathogens through recognition of pathogen-associated molecular patterns. Extracellular pathogens, such as mycobacterial lipoprotein and vesicular stomatitis virus, can be recognized by Toll-like receptors and, in turn, trigger autophagy (Xu et al., 2007; Shin et al., 2010; Nakamoto et al., 2012). Measles virus and group A Streptococcus induce functional autophagy by cell surface receptor CD46 recognition through a CD46-Cyt-1/GOPC pathway (Joubert et al., 2009). In addition, avibirnavirus VP2 is recognized by heat shock protein 90AA1 (HSP90AA1) distributed on the cell membrane and activates autophagy through the AKT/mTOR pathway (Hu et al., 2015). However, whether PRRs distributed on the cell membrane regulate autophagy upon sensing extracellular IAV is still unknown.

Heat shock protein 90AA1 is a component of the virus receptor complex and can be utilized by infectious bursal disease virus and dengue virus for entry (Reyes-Del Valle et al., 2005; Lin et al., 2007). Additionally, HSP70 and HSP90AA1 are primary receptors for bacterial lipopolysaccharide (Triantafilou et al., 2001). HSP90AB1 is a binding receptor for the Japanese encephalitis virus in Vero cells (Wang et al., 2017). Similarly, we showed that IAV attached to the cell membrane by the physical binding of HA to HSP90AA1 distributed on the cell membrane. Knockdown of HSP90AA1 or blocking it with anti-HSP90AA1 antibody greatly impaired IAV infection. Pretreatment of IAV with recombinant HSP90AA1 protein also greatly decreased infection. Moreover, we also found that IAV induced autophagy by HA binding to HSP90AA1 at the stage of attachment to the cell membrane, highlighting the role of HSP90AA1 as a PRR utilized by a broad range of viruses to induce autophagy.

## Results

### Identification of HSP90AA1 as One of the Binding Proteins of Influenza A Virus

**FIGURE 1:**
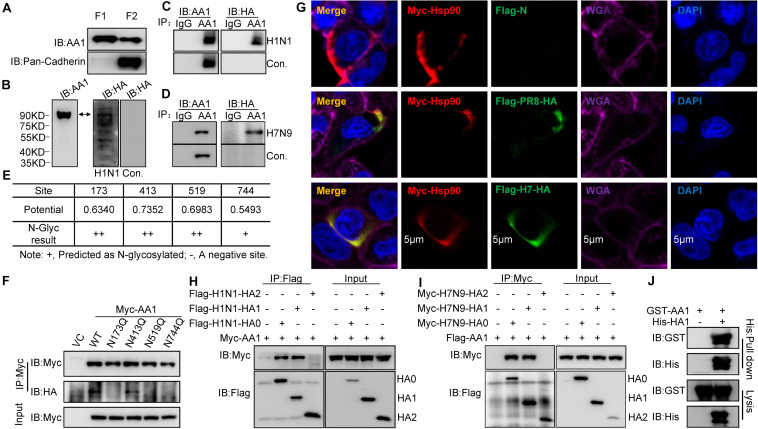
Binding of IAV HA1 to HSP90AA1. **(A)** Localization of HSP90AA1 on the membrane of A549 cells by immunoblotting analysis. Immunoblotting analysis of cytosolic proteins (F1) or membrane proteins (F2) of A549 cells was performed using anti-HSP90AA1 pAb (AA1) and anti-pan-cadherin pAb. **(B)** Membrane proteins or **(C,D)** two same anti-HSP90AA1 IP samples from A549 cells membrane components were separated on nonreducing conditions and separately transferred onto NC membranes. NC membranes were incubated in 5% skimmed in the presence or absence of **(C)** H1N1 or **(D)** H7N9 at 4°C overnights. NC membranes were then washed three times by PBS and subsequently subjected to react with the indicated rabbit anti-HA pAb or anti-HSP90AA1 (AA1) pAb. **(E,F)** N-glycosylation site of HSP90AA1 was predicted with the NetNGlyc 1.0 Server. N-glycosylation site of HSP90AA1 was predicted using the NetNGlyc 1.0 Server. Vectors expressing site-directed mutants of HSP90AA1, as indicated, were transfected into HEK293T cells for 24 h, the cells were then subjected to membrane and cytosol protein extraction. Membrane components were subjected to virus overlay protein blot assay as described in panel **(C)**. **(G)** A549 cells were transfected with Flag-HA and Myc-HSP90AA1 (Myc-AA1). Twenty-four hours later, cells were fixed and stained with anti-Flag or anti-Myc antibody, and the plasma membrane was stained with wheat germ agglutinin, and nucleus was stained with 4’,6-diamidino-2-phenylindole and then visualized by confocal microscopy (Bar = 5 μm). **(H)** HEK293T cells were transfected with the vectors expressing Flag-H1N1-HA0, Flag-H1N1-HA1, Flag-H1N1-HA2, and Myc-HSP90AA1 (Myc-AA1) or **(I)** Myc-H7N9-HA0, Myc-H7N9-HA1, Myc-H7N9-HA2, and Flag-HSP90AA1 for 48 h. Cell lysates were immunoprecipitated with anti-Flag antibodies and then analyzed by immunoblotting using the indicated antibodies. **(J)** Recombinant GST-HSP90AA1 (GST-AA1) and His-HA1 were expressed in BL21 cells and purified separately. Two proteins were incubated together and subjected to His pull-down. Immunoblotting analysis was then performed using the indicated antibodies.

Heat shock protein 90AA1 is mainly reported to be located in the cytosol and acts as a cytoplasmic chaperone. However, several studies suggested that HSP90AA1 also exists on the membrane surface and functions as a virus receptor (Eustace et al., 2004; Zuehlke et al., 2015; Garcia et al., 2016). To demonstrate that A549 cell surfaces have HSP90AA1, immunoblotting analysis of HSP90AA1 was performed in cell membrane samples by using an anti-HSP90AA1 antibody and an anti-pan-cadherin antibody as a plasma membrane marker. As shown in Figure 1A, HSP90AA1 could be recognized by the anti-HSP90AA1 antibody in the membrane component obtained from A549 cells.

Heat shock protein 90AA1 has been shown to serve as a viral receptor (Reyes-Del Valle et al., 2005; Lin et al., 2007). To determine whether IAV binds to cell surface HSP90AA1, HSP90AA1 was immunoprecipitated from membrane components by using an anti-HSP90AA1 antibody, the membrane proteins, or immunoprecipitation (IP) samples were separated by sodium dodecyl sulfate-polyacrylamide gel electrophoresis (SDS-PAGE) and transferred to nitrocellulose (NC) membrane, then virus overlay protein blot assay was carried out. As expected, the immunoblotting analysis showed that HSP90AA1 could be recognized by using either anti-HSP90AA1 pAb or anti-HA pAb on the NC membrane in the presence of H1N1 or H7N9 virus, while only by anti-HSP90AA1 pAb, not anti-HA antibody in the absence of H1N1 or H7N9 virus (Figures 1B–D), suggesting that HA of H1N1 or H7N9 could bind to membrane HSP90AA1. We then predicted the N-glycosylation site on HSP90AA1 using the NetNGlyc 1.0 Server and accordingly constructed the vectors expressing HSP90AA1 mutants, N173Q, N413Q, N519Q, and N744Q (Figure 1E). As shown in Figure 1F, N173Q, N519Q, and N744Q, but not N413Q, exhibited largely weaker binding to the virus, suggesting that glycosylation on N173, N519, or N744 is important for binding of IAV to HSP90AA1. Next, as shown in Figure 1G, H1N1 or H7N9 HA colocalized with HSP90AA1 that was distributed on the cell membrane. Moreover, anti-Flag IP showed that HA0 and HA1, but not HA2, interacted with HSP90AA1 (Figures 1H,I), suggesting that HA1 is responsible for binding to HSP90AA1. Similarly, as shown in Figure 1J, recombinant His-tagged HA1 pulled down glutathione S-transferase (GST)-tagged HSP90AA1 *in vitro*, suggesting a direct interaction between HA1 and HSP90AA1.

Overall, these results indicate that HSP90AA1 is one of the proteins on A549 cells, which can bind to IAV HA1 protein, the main receptor-recognition protein of IAV.

### Anti-heat Shock Protein 90AA1 Polyclonal Antibody and Recombinant Heat Shock Protein 90AA1 Protein Inhibit Influenza A Virus Infection in A549 Cells

**FIGURE 2:**
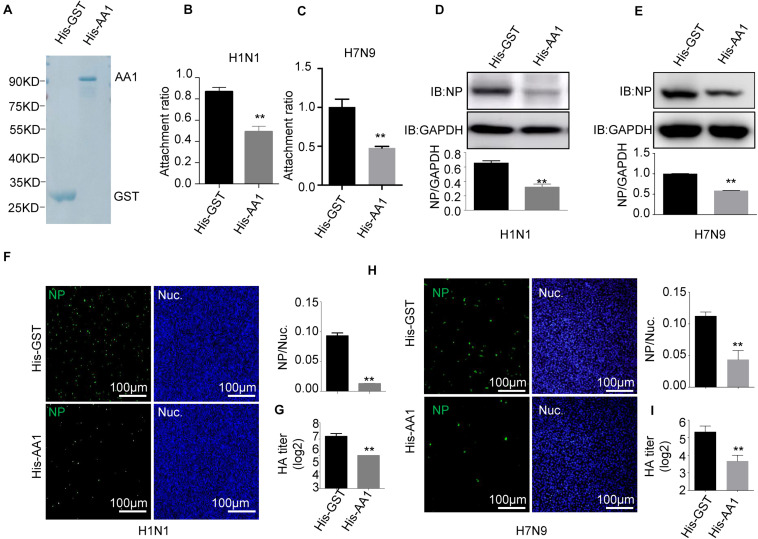
Recombinant HSP90AA1 protein blocks entry of IAV. **(A)** His-HSP90AA1 (His-AA1) and His-GST were purified and separated using SDS-PAGE and stained with Coomassie brilliant blue. Infection inhibition assays with HSP90AA1 in A549 cells. **(B)** H1N1 or **(C)** H7N9 was pretreated with His-HSP90AA1 (His-AA1) or His-GST at 37°C for 1 h and then added into chilled A549 cells for 2 h at 4°C. Cells were then incubated at 37°C for 1 h. Attachment ratio of H1N1 and H7N9 viruses was quantified by measuring *NP* levels through qPCR. **(D)** H1N1 or (E) H7N9 was pretreated with His-HSP90AA1 (His-AA1) or His-GST at 37°C for 1 h and then infected A549 cells for another 12 h at 37°C after attachment of the virus to A549 cells. Cells were lysed and subjected to immunoblotting analysis by using indicated antibodies. **(F,H)** After infection of the virus to A549 cells as described in panels **(D,E)**, the cells were then fixed. Confocal analysis was then performed. **(G,I)** After attachment of the virus to A549 cells as described in panels **(D,E)**, cells were cultured in DMEM for another 48 h, the cell culture supernatant was harvested, a standard hemagglutination assay determined virus titers in supernatants. Error bars: mean ± SD of three independent tests. Two-way ANOVA; *P < 0.05; **P < 0.01 compared with control.

To further verify that HSP90AA1 is a functional part of the process of virus entry, we assessed the role of HSP90AA1 on virus attachment. Vectors expressing recombinant His-HSP90AA1 and His-GST were constructed, and proteins were then expressed and purified (Figure 2A). Influenza A virus, H1N1, or H7N9 were pretreated with purified either His-HSP90AA1 or control His-GST at 37°C for 1 h. Next, the virus–protein mix [multiplicity of infection (MOI) = 1.0] was added into the chilled cells for 2 h at 4°C, the unattached viruses were washed away with cold phosphate-buffered saline (PBS), and cells were then incubated at 37°C for 1 h, and after that, cells were subjected to RNA isolation. Quantitative reverse transcription PCR (RT-qPCR) of the NP gene measured the RNA copies of the attached virus. Comparative analysis of *NP* RNA copies in the His-HSP90AA1-treated samples and the His-GST-treated cells showed that treatment of His-HSP90AA1 proteins decreased virus attachment to cells (Figures 2B,C). Next, to test whether HSP90AA1 is critical for IAV infection, immunofluorescence assay (IFA) and immunoblotting analysis were performed. As shown in Figures 2D,E, compared with the pretreatment of H1N1 or H7N9 with His-GST, pretreatment with recombinant HSP90AA1 protein greatly reduced the level of NP. Consistently, the number of H1N1- and H7N9-positive cells with green fluorescence significantly decreased in cells infected with virus pretreated with His-HSP90AA1 protein (Figures 2F,H) compared with their control treatments, respectively. Moreover, the virus growth kinetics measured by hemagglutination assay showed that pretreating virus with the recombinant HSP90AA1 protein reduced the virus production in A549 cells (Figures 2G,I).

**FIGURE 3:**
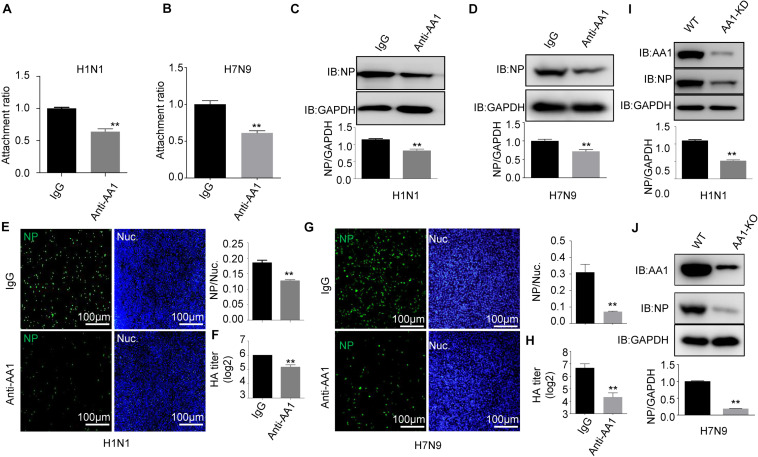
Blocking A549 cells with anti-HSP90AA1 antibody or knocking down of HSP90AA1 inhibits IAV infection. **(A,B)** A549 cells were incubated with DMEM in the presence of anti-HSP90AA1 antibodies (anti-AA1) or control IgG at 4°C for 1 h and then were inoculated with **(A)** H1N1 or **(B)** H7N9 for 2 h at 4°C. Attachment ratio of H1N1 and H7N9 viruses were quantified by measuring *NP* levels through qPCR. **(C,D)** A549 cells were incubated with DMEM in the presence of anti-HSP90AA1 antibodies (anti-AA1) or control IgG at 4°C for 1 h and then infected A549 cells with **(C)** H1N1 or **(D)** H7N9 for another 12 h at 37°C after attachment of the virus to A549 cells. Cells were lysed and subjected to immunoblotting analysis by using indicated antibodies. **(E,G)** After infection of the virus to A549 cells as described in panels **(C,D)**, cells were then fixed. Confocal analysis was then performed. **(F,H)** After attachment of the virus to A549 cells as described in panels **(C,D)**, cells were cultured in DMEM for another 48 h, cell culture supernatant was harvested, and a standard hemagglutination assay determined virus titers in supernatants. **(I,J)** Western blotting analysis of NP levels after virus infection for 12 h in wild type or HSP90AA1-knockdown (AA1-KD) cells. Error bars: mean ± SD of three independent tests. Two-way ANOVA; *P < 0.05; **P < 0.01 compared with control.

In parallel, A549 cells were preblocked with anti-HSP90AA1 or control anti-immunoglobulin G (IgG) antibodies at 4°C for 1 h and then chilled at 4°C, followed by the addition of H1N1 or H7N9 for 2 h at 4°C. These cells were then washed, incubated at 37°C for 1 h, lysed, and prepared for RT-qPCR. As shown in Figures 3A,B, the treatment of the anti-HSP90AA1 antibody also displayed the decrease of the virus attachment to cells. Then, IFA and immunoblotting analysis were performed. The level of NP decreased greatly in cells preblocked with anti-HSP90AA1 pAb compared with IgG pAb (Figures 3C,D). The number of H1N1- and H7N9-positive cells with green fluorescence significantly decreased in cells infected with virus preblocked with an anti-HSP90AA1 pAb compared with the IgG (Figures 3E,G). The virus growth kinetics measured by hemagglutination assay showed that preblocking cells with anti-HSP90AA1 pAb reduced the virus production in A549 cells (Figures 3F,H). Furthermore, NP levels decreased greatly in the HSP90AA1 knockdown A549 cell lines (Figures 3I,J), confirming that HSP90AA1 was critical for IAV infection.

Collectively, the results demonstrate that HSP90AA1 distributed on the cell membrane plays a critical role in the entry and infection of IAV.

### HA1 Is Critical for the Induction of Autophagy

**FIGURE 4:**
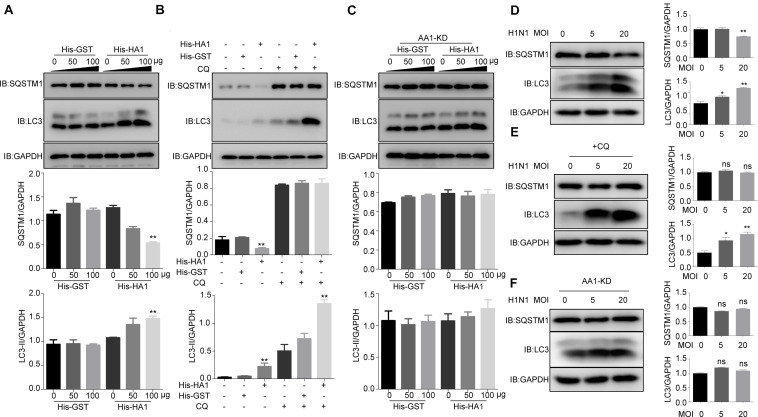
IAV HA1 induces autophagy through HSP90AA1. **(A)** Immunoblotting analysis of autophagy was analyzed; A549 cells were cultured in DMEM with 10% FBS in the presence of His-GST or His-HA1 protein as indicated for 2 h. **(B)** A549 cells treated with and without chloroquine for 2 h, then treated with His-GST or His-HA1. Immunoblotting analysis of autophagy flux was detected by using the indicated antibodies. **(C)** HSP90AA1-knockdown (AA1-KD) A549 cells were cultured in DMEM with 10% FBS in the presence of His-GST or His-HA1 protein as indicated for 2 h. Immunoblotting analysis was performed by using the indicated antibodies. **(D)** A549 cells were incubated with H1N1 at 4°C for 2 h (MOI = 5.0 or 20.0), and cell lysates were analyzed by Western blotting with the indicated antibodies. **(E)** A549 cells were treated with chloroquine for 2 h, then incubated with H1N1 at 4°C for 2 h. Immunoblotting analysis was performed by using the indicated antibodies. **(F)** HSP90AA1-knockdown (AA1-KD) A549 cells were incubated with H1N1 at 4°C for 2 h (MOI = 5.0 or 20.0), and cell lysates were analyzed by Western blotting with the indicated antibodies. Error bars: mean ± SD of three independent tests. Two-way ANOVA; *P < 0.05; **P < 0.01 compared with control.

Because HSP90AA1 has been reported to induce autophagy when it recognizes the pathogen, we next tested whether HA1 treatment promoted autophagy. As shown in Figure 4A, HA1 treatment, but not GST treatment, increased the level of LC3-II and decreased the level of SQSTM1 significantly in a dose-dependent manner. Interestingly, LC3-II level increased significantly in the presence of chloroquine, suggesting that HA1-induced increase of LC3-II resulted from newly formed lipidation of LC3 but not from inhibition of LC3-II degradation (Figure 4B). However, knockdown of HSP90AA1 counteracted the effect of HA1 on the levels of LC3-II and SQSTM1 (Figure 4C). Further study showed that incubation cells with the H1N1 at 4°C increased the level of LC3-II and decreased the level of SQSTM1 significantly (Figure 4D), and the LC3-II level increased significantly in the presence of chloroquine (Figure 4E), suggesting that H1N1 promoted autophagy at attachment stage. However, the downregulation of HSP90AA1 counteracted the effect of H1N1 on the levels of LC3-II and SQSTM1 (Figure 4F). Collectively, the results mentioned earlier suggest that HSP90AA1 is required for the HA1 of H1N1 IAV-mediated induction of autophagy at the attachment stage.

### HA1 Binding to Heat Shock Protein 90AA1 Inhibited the Phosphorylation of mTOR and AKT

**FIGURE 5:**
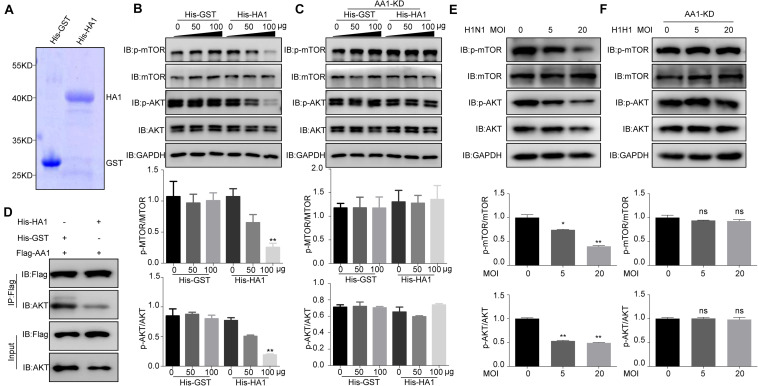
IAV HA1 is critical for dephosphorylation of AKT/mTOR. **(A)** Purified His-tagged HA1 was separated using SDS-PAGE and stained with Coomassie brilliant blue. **(B)** A549 cells and **(C)** HSP90AA1-knockdown (AA1-KD) A549 cells were serum-starved for 12 h and then incubated in DMEM containing His-GST or His-HA1 protein as indicated for 2 h. Cell lysates were analyzed by immunoblotting with anti-p-mTOR, anti-p-AKT, anti-mTOR, anti-AKT, and anti-glyceraldehyde 3-phosphate dehydrogenase antibodies. Ratio of p-mTOR to mTOR or p-AKT to AKT was normalized to control conditions. **(D)** HEK293T cells were transfected with Flag-HSP90AA1 (Flag-AA1). After transfection for 36 h, cells were treated with His-GST or His-HA1 (100 μg) for 12 h. Then, Flag-tagged sample was immunoprecipitated and immunoblotted with the indicated antibodies. **(E)** A549 cells or **(F)** HSP90AA1-knockdown (AA1-KD) A549 cells were incubated with H1N1 influenza A virus (MOI = 5.0 or 20.0) at 4°C for 2 h. Cell lysates were analyzed by immunoblotting with the indicated antibodies. Ratio of p-mTOR to mTOR or p-AKT to AKT was analyzed as **(B,C)**. Error bars: mean ± SD of three independent tests. Two-way ANOVA; *P < 0.05; **P < 0.01 compared with control.

Given that HA1 is capable of binding to cell surface HSP90AA1, we want to determine whether HA1 is involved in the AKT/mTOR pathway. The recombinant proteins His-HA1 and His-GST were expressed and purified (Figure 5A). A549 cells were then incubated with the purified His-HA1 or His-GST for 2 h, and the phosphorylation level of mTOR and AKT were tested. As shown in Figure 5B, the phosphorylation levels of mTOR and AKT were decreased in His-HA1-treated A549 cells in a dose-dependent manner. However, HSP90AA1 knockdown counteracted the effects of HA1 on inhibiting phosphorylation of mTOR and AKT (Figure 5C), suggesting that HSP90AA1 was required for HA1-mediated inhibition of mTOR and AKT phosphorylation. A previous study showed that the HSP90AA1 interaction with AKT was required for phosphorylation of AKT (Sato et al., 2000), and crosslinking of cell surface HSP90AA1 was sufficient to inactivate AKT/mTOR pathway (Xiao et al., 2018). Thereby, we determined whether HA1 binding to HSP90AA1 inhibited the interaction between HSP90AA1 and AKT. Co-IP showed that HA1 treatment indeed blocked the binding of HSP90AA1 to AKT (Figure 5D). We then investigated the role of IAV on the AKT/mTOR pathway. For the purpose, A549 cells were incubated with the H1N1 virus at 4°C for 2 h, and the phosphorylation levels of mTOR and AKT were tested by Western blotting. As shown in Figure 5E, the phosphorylation levels of mTOR and AKT were decreased in H1N1-infected A549 cells. However, the downregulation of HSP90AA1 counteracted the effects of HA1 on inhibiting phosphorylation of mTOR and AKT (Figure 5F). Collectively, the results indicated that the HA1 of IAV blocked the AKT/mTOR pathway by inhibiting the interaction between HSP90AA1 and AKT.

## Discussion

In recent years, a few reports have shown HSP90AA1 on the cell surface (Tsutsumi and Neckers, 2010; Wei et al., 2014), although it predominantly localizes to the cytoplasm. In this study, we demonstrated that HSP90AA1 could be extracted from the cell membrane and reacted with IAV. Subsequent evidence, such as the observation of the surface localization of HSP90AA1 and IAV HA1 (Figure 1C), and the results that blocking cell surface HSP90AA1 with anti-HSP90AA1 antibodies and treating H1N1 viruses with purified HSP90AA1 protein both substantially inhibited attachment of virus on the cell membrane and reduced virus infection greatly (Figure 2), confirming that HSP90AA1 participated in the process of influenza virus attachment.

Virus receptors on the cell surface are critical for virus entry (Mukherjee et al., 2018). It is generally accepted that SA molecules on the host cell surface are the main receptor for IAV. However, there is still debate as to whether IAV requires additional host factors for successful attachment and entry into target cells. In recent years, scientists have found other molecules, such as DC-SIGN, FFAR2, and EPN1, on the host cell surface assisting in the entry of IAV (Chen and Zhuang, 2008; Wang et al., 2008; Shin et al., 2010; Londrigan et al., 2011; Wang et al., 2020). Fibronectin is required for the α-2,6-linkage SA-binding preference of IAVs (Horasis et al., 2012). Nucleolin, one of the host cell surface proteins, is required for the entry of multiple IAVs (Chan et al., 2016). Additionally, the receptor-binding domain of IAV HA is HA1. Influenza A virus HA binds to SAs through HA1, which initiates the entry of IAV. Interestingly, in the present study, IAV HA binds to HSP90AA1 through HA1 as well, suggesting that the HSP90AA1 on cell surface ought to be a component of the IAV receptor complex. However, whether HSP90AA1 assists in the binding of the α-2,6-linkage SA to IAV HA1 or is a receptor of IAV independent of SA needs for further investigation.

Many studies have discussed the relation between IAV and autophagy. For example, H1N1 infection induces autophagy, which benefits H1N1 replication (Yeganeh et al., 2018). A recent study showed that IAV replication requires an autophagy pathway to enhance viral RNA synthesis via the interaction of PB2 and HSP90AA1 by modulating HSP90AA1 expression and the AKT-mTOR signaling pathway in host cells (Wang et al., 2019). The viral proteins HA, M2, and NS1 are involved in initiating the formation of autophagosomes in infected cells (Zhirnov and Klenk, 2013; Liu et al., 2016). Interestingly, our study showed that autophagy was induced upon attachment of IAV HA to HSP90AA1, which in turn induces autophagy by inactivating the AKT-mTOR pathway. Moreover, IAV infection induced complete autophagy at the early stage of IAV infection through the binding of HA1 to HSP90AA1, implying that autophagy might be used by IAV to degrade other innate immunity factors or facilitate IAV entry by other unknown mechanisms.

HSP90AA1 is a multifunctional protein. HSP90 isoforms include the well-known HSP90AA1 and HSP90AB1 (Zuehlke et al., 2015); they have distinct functions in cells. Previous studies have suggested that HSP90AA1 possibly acts as a molecular chaperone and is involved in the assembly and nuclear transport of viral RNA polymerase subunits, followed by the formation of a mature ternary polymerase complex (Tadasuke et al., 2007). Recently, Wang et al. (2019) showed that IAV NP and M2 induce the expression of HSP90AA1 and subsequently enhance viral RNA synthesis partially through the interaction of PB2 and HSP90AA1. It is therefore not surprising that HSP90AA1 may play a role in multiple stages of the influenza viral life cycle.

## Materials and Methods

### Viruses and Cells

Laboratory influenza virus H1N1 (A/Puerto Rico/8/34) (referred to H1N1 in this study) and H7N9 (A/Hangzhou/1/2013) was propagated in 7- to 9-day-old embryonated chicken eggs. HEK293T and A549 cells were cultured in Dulbecco’s modified Eagle’s medium (DMEM; Gibco, Thermo Fisher Scientific, United States) supplemented with 10% serum at 5% carbon dioxide at 37°C.

### Antibodies and Reagents

Mouse anti-NP and anti-His antibodies were maintained in our lab. The anti-HA H1 antibody (ab91531) was purchased from Abcam, and the mouse anti-HA H7 antibody was maintained in this lab. Rabbit polyclonal antibodies anti-Myc (R1208-1), anti-glyceraldehyde 3-phosphate dehydrogenase (EM1101), were purchased from Huaan Biological Technology. An anti-Myc tag mAb, clone 9E10 (05-419), was purchased from Millipore. Rabbit anti-HSP90AA1 (13171-1-AP) antibody was purchased from Proteintech. Rabbit IgG (A7016) was purchased from Beyotime. Nickel–nitrilotriacetic acid agarose beads (160028558) were obtained from Qiagen. Horseradish peroxidase (5220-0336)-labeled and fluorescein (5230-0427)-labeled goat anti-mouse antibodies were purchased from KPL (Milford, MA). Alexa Fluor 488-labeled anti-chicken IgY (Abcam, ab150169) and Alexa Fluor 546-conjugated anti-mouse and anti-rabbit IgG (Invitrogen, United States) were also used in this study. Wheat germ agglutinin conjugates (29026-1) was purchased from Biotium.

### Plasmid Construction

The open reading frame (ORF) of *HSP90AA1* was cloned from the complementary DNA (cDNA) of A549 cells. For Myc-HSP90AA1, forward primer (5′-GCGTCGAC CATGCCTGAGGAAACCCAGACCCAAG-3′) and reverse primer (5′-GGTACCTTAGTCTACTTCTTCCATGCGTGAT-3′) were used to generate full-length *HSP90AA1* in pCMV-Myc vector with N-terminal in frame with Myc sequence resulting in Myc-HSP90AA1. The N-glycosylation site was predicted with the NetNGlyc 1.0 Server, and the predicted sites were N173, N413, N519, and N744. The mutants N173Q, N413Q, N519Q, and N744Q were constructed with the site-mutation primers. The forward primers N173Q (5′-GAGAGA GCTCATTTCACAGTCATCAGATGCATTG-3′), N413Q (5′-GATCAAGAAGAGCTCCAGAAAACAAAGCCCATC-3′), N51 9Q (5′-GAGGATCTCCCTCTACAGATATCCCGTGAGATG-3′), and N744Q (5′-CAAGCCCTAAGAGACCAGTCAACAATG GGTTAC-3′) and the reverse-primers were reverse complemented. H1N1 *HA* fragment was amplified from H1N1 virus cDNA with a forward primer (5′-GCGTCGACCATGGA CACAATATGTATAGGCTACC-3′) and a reverse primer (5′- GGGGTACCTCAGATGCATATTCTGCACTGCAAA-3′), *HA1* with a forward primer (5′-GCGTCGACCATGGACACAATAT GTATAGGCTACC-3′) and a reverse primer (5′-GGGGTACCTC ATCTGGATTGAATGGACGGAATG-3′), *HA2* with a forward primer (5′-GCGTCGACCATGGGTCTATTTGGAGCCATTGC CG-3′) and a reverse primer (5′-GGGGTACCTCAGATGCA TATTCTGCACTGCAAA-3′) cloned into pCMV-Flag vector with N-terminal in frame with Flag sequence resulting in Flag-H1N1-HA0, Flag-H1N1-HA1, and Flag-H1N1-HA2. For H7N9, the *HA* fragment was amplified from H7N9 virus cDNA with a forward primer (5′-GCGTCGACGATGGACAAAATCTGCCTC GGAC-3′) and a reverse primer (5′-GCGGTACCTTATATACA AATAGTGCAC-3′), *HA1* with a forward primer (5′-GCG TCGACGATGGACAAAATCTGCCTCGGAC-3′) and a reverse primer (5′-GCGGTACCCTATCTTCCCTTTGGAATCTC-3′), *HA2* with a forward primer (5′-GCGTCGACGATGGGCC TATTTGGTGCTATAG-3′) and a reverse primer (5′-GCG GTACCTTATATACAAATAGTGCAC-3′) cloned into pCMV-Flag vector with N-terminal in frame with Flag sequence resulting in Flag-H7N9-HA0, Flag-H7N9-HA1, and Flag-H7N9-HA2.

### Co-immunoprecipitation Assay

HEK293T cells were transfected with plasmid as indicated and empty vectors for 48 h and then lysed using NP40 lysis buffer. Cell lysates were incubated with anti-Flag or anti-Myc antibody and protein A/G beads for 4 h at 4°C. After centrifugation, the supernatants were removed, and the pellets were resuspended in washing buffer. Centrifugation and resuspension of the pellets in fresh washing buffer were performed five times. Finally, the pellets were lysed in lysis buffer for immunoblotting analysis.

### Confocal Microscopy

Confocal microscopy was used to observe the colocalization of HA and HSP90AA1. A549 cells were cotransfected with Flag-HA and Myc-HSP90AA1; Flag-N was used as a negative control. Twenty-four hours post-transfection, the samples were fixed in 4% paraformaldehyde for 15 min at room temperature, without permeabilized cells, were then incubated with anti-Flag and anti-Myc antibodies for 2 h at 37°C, and finally incubated with the appropriate secondary antibodies for 1 h at 37°C. Cells were stained with wheat germ agglutinin as the product instruction and were then stained with 4′,6-diamidino-2-phenylindole. After that, the cells were scanned with a Nikon A1R/A1 laser scanning confocal microscope.

### Subcellular Proteins Extraction of A549 Cells

Differential extraction of A549 cells using ProteoExtract Subcellular Proteome Extraction Kit (Calbiochem, 539790) was performed according to the manufacturer’s protocol. Briefly, A549 cells were collected and washed two times with wash buffer. We mixed a 1-ml extraction buffer I and a 5-μl protease inhibitor cocktail and immediately incubated cells for 10 min at 4°C with gentle rocking. The supernatant (cytosolic protein extraction: fraction (1) was transferred to a clean tube. Then, we added a 1-ml extraction buffer II and a 5-μl protease inhibitor cocktail to the remaining cellular material and incubated for 30 min at 4°C with gentle agitation. The supernatant (membrane/organelle protein fraction: fraction (2) was transferred to a clean tube. Different fractions from A549 were separated by SDS-PAGE and transferred to NC membrane for blotting with the indicated antibodies.

### Immunoprecipitation of Heat Shock Protein 90AA1 and Virus Overlay Protein Blot Assay

Equal amounts of the membrane components from A549 cells were precipitated by IgG or anti-HSP90AA1 antibody-coated beads. The samples were separated by SDS-PAGE under nonreducing conditions using 8% gels and transferred on to a NC membrane. A virus overlay protein blot assay was performed to verify that the isolated proteins are involved in virus binding. Briefly, membranes were blocked with 5% skimmed milk for 1 h at 37°C and then washed with PBS three times, subsequently incubated overnight with or without 3 × 10^6^ PFU of the H1N1 virus in PBS with 5% skimmed milk at 4°C. After being washed three times with PBS, the membrane was incubated with anti-IAV HA H1 antibody overnight at 4°C and then incubated with a peroxidase-labeled goat polyclonal anti-rabbit IgG antibody in 5% skimmed milk for 1 h as a secondary antibody at room temperature. After washing with PBS-Tween five times, chemiluminescence was performed using a Femto-Mol detection system (Pierce) to detect the antigen. In parallel, membrane protein samples were also reacted with anti-HSP90AA1 antibody alone.

### Protein Expression and Purification

Polymerase chain reaction amplified the ORF of human HSP90AA1 from A549 cDNA, and the ORF of *HA1* was amplified from H1N1 virus cDNA. They were all cloned into the pET-28a vector, which includes a His_6_ tag, and the *HSP90AA1* was also cloned into the pGEX-4T-1 vector, which includes a GST tag. These constructs were used to transform *Escherichia coli* BL21 for expression. For His_6_ tag protein purification, the washed bacterial pellets were resuspended in lysis buffer [50-mM Tris-base, 150-mM sodium chloride (NaCl), 1-mM phenylmethylsulfonyl fluoride]. After sonication and a freeze–thaw step, the supernatant was incubated with nickel–nitrilotriacetic acid resin in the presence of 10-mM imidazole. Beads were washed with gradually increased concentrations of imidazole. Purified proteins were eluted in lysis buffer containing 100-mM imidazole. Glutathione S-transferase tag protein was purified with glutathione resin, beads were washed four times with 30-ml lysis buffer, and purified proteins were eluted in lysis buffer containing 1 mg/ml reduced glutathione. The concentrations of the purified proteins were measured with the bicinchoninic acid assay.

### Pull-Down Assay by Immunoprecipitation

Five hundred micrograms of purified protein Gst-HSP90AA1 and His-HA1 was gently mixed for 4 h at 4°C. Eighty microliters of Ni-NTA resin beads were then added. The mixture was gently mixed for 4–6 h at 4°C and centrifuged at 1,000 × *g* for 5 min to collect the pellet. Nonspecific binding to the resin was avoided by washing the pellet with 10 volumes of the native binding buffer [50-mM Tris-base, 150-mM NaCl (pH 7.8)] and was followed by 10 volumes of native wash buffer 1 [50-mM Tris-base, 150-mM NaCl (pH 7.2)]. To remove the more nonspecific binding, the pellet was washed with 10 volumes of native wash buffer 2 and 3 [50-mM Tris-base, 150-mM NaCl (pH 6.8 and 6.3)]. The final pellet was suspended in 30–40 μl of sample buffer, heated to 95°C for 10 min, and then centrifuged at 12,000 × *g* for 10 min to remove the beads. Pull-down eluates were analyzed by SDS-PAGE and immunoblotting with anti-His and anti-GST antibodies as described.

### Infection Inhibition by Anti-heat Shock Protein 90AA1 Antibody and Recombination Protein Heat Shock Protein 90AA1

To detect the attachment of H1N1 and H7N9, viruses were incubated with 100-μg recombinant HSP90AA1 protein at 37°C for 1 h, or A549 cells were incubated with anti-HSP90AA1 antibody (5 μg/ml) at 4°C for 1 h, then H1N1 or H7N9 (MOI = 1.0) was added to cells for attachment at 4°C for 2 h, and cells were washed with PBS for three times. The cells were then incubated at 37°C for 1 h and then lysed with trizol, prepared for RT-qPCR.

Immunofluorescence assay and immunoblotting analysis were performed to detect the infection of H1N1 and H7N9. A549 cells were seeded in 12-well plates with DMEM supplemented with 10% FBS for 5 h. The medium was then removed. Additionally, MOI of 0.1 of H1N1 or H7N9 virus was incubated with recombinant His-HSP90AA1 protein (100 μg) at 37°C for 1 h before infection. These HSP90AA1-containing viral mixtures were used to infect A549 cells seeded in 12-well plates for 1 h. Similarly, His-GST was used as a control. Later, infected cells were washed three times with a culture medium. Fresh medium was then added to the infected cells, and infection was allowed to proceed for 12 h at 37°C. After infection, the cell lysates were collected, and the virus protein NP level was detected to examine the amount of IAV. Also, the same process was carried out except that the infection time was changed to proceed for 48 h at 37°C. After infection, the supernatant was collected, and a standard hemagglutination assay measured the viral HA titer.

A549 cells were incubated with rabbit anti-HSP90AA1 pAb (5 μg/ml) at 4°C for 1 h. Similarly, the cells were treated with similar concentrations of rabbit IgG as a control. After the media were removed, the cells were infected with H1N1 (MOI = 0.1) in a serum-free medium for 1 h at 4°C and then washed three times with fresh culture medium. Fresh medium was then added to the infected cells, and the infection was allowed to proceed for 12 h. After infection, cell lysates were collected to detect the virus protein NP level. The same process was carried out except that the infection time was changed to proceed for 48 h at 37°C. After infection, the cell culture supernatant was harvested; a standard hemagglutination assay determined virus titers in supernatants.

### CRISPR/Cas9 for Heat Shock Protein 90AA1 Construction and Transfection

CRISPR/Cas9 genomic editing for gene knockdown was used as previously described (Norouzi-Barough et al., 2018). Small guide RNA was cloned into the vector lentiCRISPRv2 (Addgene) and transfected into A549 cells. Twenty-four hours after transfection, cells were placed under puromycin selection for 1 week, and single clones were picked, grown, and identified by immunoblot analysis.

To create gene-targeted alleles encoding HSP90AA1 in human cells, CRISPR guide RNA sequence was designed based on its specificity scores using the CRISPR DESIGN tool. The small guide RNA sequence was cloned into the pSpCas9 (BB)-2A-Puro plasmid (pX459, Addgene). The following guide RNA sequence was then used:

HSP90AA1-sg1: 5′-CGGGTATTCAGCACTCTGGG-3′

### Induction of Autophagy by Virus or HA1 Stimulation

Wild type or HSP90AA1 knockdown A549 cell lines were cultured in 12-well plates. Next, cells were cultured in serum-free DMEM for 12 h at 37°C and then incubated with H1N1 virus (MOI = 5.0 or 10.0) or recombinant proteins His-HA1 and His-GST (control) for 2 h at 4°C. The cells were harvested, and AKT and mTOR levels were detected. Similarly, His-HA1 and His-GST (control) were incubated with wild type and HSP90AA1 knockdown A549 cells at 4°C for 2 h; the autophagy flux was detected by treating with chloroquine. The cells were harvested and immunoblotted by using anti-SQSTM1 and anti-LC3 antibodies to monitor autophagy.

### Quantification and Statistical Analysis

Image J was used for the quantification of band intensity for immunoblots. The GraphPad Prism software (GraphPad Software, Inc., La Jolla, CA, United States) was used for all the statistical analyses with the student’s test. The *P*-values in the figures are defined as follows: ^∗∗^*P* < 0.01; ^∗^*P* < 0.05; and ns (nonsignificant), *P* > 0.05.

## Data Availability Statement

All datasets presented in this study are included in the article.

## Author Contributions

BH, JZ, XW, and TZ conceived and designed the experiments and participated in the discussion. XW, TZ, LL, and XP performed the experiments. XW, TZ, YZ, JL, and YY analyzed the data. JZ, BH, XW, and TZ wrote the manuscript. All authors have read and agreed to the published version of the manuscript.

## Conflict of Interest

The authors declare that the research was conducted in the absence of any commercial or financial relationships that could be construed as a potential conflict of interest.

## References

[B1] Chan C. M. Chu H. Zhang A. J. Leung L. H. Sze K. H. Kao R. Y. (2016). Hemagglutinin of influenza A virus binds specifically to cell surface nucleolin and plays a role in virus internalization. *Virology* 494 78–88. 10.1016/j.virol.2016.04.008 27085069

[B2] Chen C. Zhuang X. (2008). Epsin 1 is a cargo-specific adaptor for the clathrin-mediated endocytosis of the influenza virus. *Proc. Natl. Acad. Sci. U S A.* 105 11790–11795. 10.1073/pnas.0803711105 18689690 PMC2504482

[B3] Eustace B. K. Sakurai T. Stewart J. K. Yimlamai D. Unger C. Zehetmeier C. (2004). Functional proteomic screens reveal an essential extracellular role for hsp90 alpha in cancer cell invasiveness. *Nat. Cell Biol.* 6 507–514. 10.1038/ncb1131 15146192

[B4] Fukuzawa K. Omagari K. Nakajima K. Nobusawa E. Tanaka S. (2011). Sialic acid recognition of the pandemic influenza 2009 H1N1 virus: binding mechanism between human receptor and influenza hemagglutinin. *Prot. Pept. Lett.* 18 530–539. 10.2174/092986611794927893 21235490

[B5] Garcia R. Merino D. Gomez J. M. Nistal J. F. Hurle M. A. Cortajarena A. L. (2016). Extracellular heat shock protein 90 binding to TGFbeta receptor I participates in TGFbeta-mediated collagen production in myocardial fibroblasts. *Cell Signal* 28 1563–1579. 10.1016/j.cellsig.2016.07.003 27418101

[B6] Hers J. F. (1966). Disturbances of the ciliated epithelium due to influenza virus. *Am. Rev. Respir. Dis.* 93 162–177. 10.1164/arrd.1966.93.3P2.162 5333643

[B7] Horasis S. Y. Leung O. T. W. L. Renee W. Y. (2012). Entry of Influenza A Virus with a α2,6-Linked Sialic Acid Binding Preference Requires Host Fibronectin. *J. Virol.* 89 10704–13. 10.1128/jvi.01166-12 22837202 PMC3457276

[B8] Hu B. Zhang Y. Jia L. Wu H. Fan C. Sun Y. (2015). Binding of the pathogen receptor HSP90AA1 to avibirnavirus VP2 induces autophagy by inactivating the AKT-MTOR pathway. *Autophagy* 11 503–515. 10.1080/15548627.2015.1017184 25714412 PMC4502722

[B9] Joubert P. E. Meiffren G. Gregoire I. P. Pontini G. Richetta C. Flacher M. (2009). Autophagy induction by the pathogen receptor CD46. *Cell Host Microbe.* 6 354–366. 10.1016/j.chom.2009.09.006 19837375

[B10] Kudchodkar S. B. Levine B. (2009). Viruses and autophagy. *Rev. Med. Virol.* 19 359–378. 10.1002/rmv.630 19750559 PMC2852112

[B11] Lin T. W. Lo C. W. Lai S. Y. Fan R. J. Lo C. J. Chou Y. M. (2007). Chicken heat shock protein 90 is a component of the putative cellular receptor complex of infectious bursal disease virus. *J. Virol.* 81 8730–8741. 10.1128/JVI.00332337 17522206 PMC1951386

[B12] Liu G. Zhong M. Guo C. Komatsu M. Xu J. Wang Y. (2016). Autophagy is involved in regulating influenza A virus RNA and protein synthesis associated with both modulation of Hsp90 induction and mTOR/p70S6K signaling path- way. *Int. J. Biochem. Cell Biol.* 72 100–108. 10.1016/j.biocel.2016.01.012 26794463

[B13] Londrigan S. L. Turville S. G. Tate M. D. Deng Y. M. Brooks A. G. Reading P. C. (2011). N-linked glycosylation facilitates sialic acid-independent attachment and entry of influenza A viruses into cells expressing DC-SIGN or L-SIGN. *J. Virol.* 85 2990–3000. 10.1128/JVI.017051710 21191006 PMC3067946

[B14] Mukherjee S. Sengupta N. Chaudhuri A. Akbar I. Singh N. Chakraborty S. (2018). PLVAP and GKN3 Are Two Critical Host Cell Receptors Which Facilitate Japanese Encephalitis Virus Entry Into Neurons. *Sci. Rep.* 8:11784. 10.1038/s41598-018-30054-z 30082709 PMC6079088

[B15] Nakamoto M. Moy R. H. Xu J. Bambina S. Yasunaga A. Shelly S. S. (2012). Virus recognition by Toll-7 activates antiviral autophagy in Drosophila. *Immunity* 36 658–667. 10.1016/j.immuni.2012.03.003 22464169 PMC3334418

[B16] Norouzi-Barough L. Sarookhani M. Salehi R. Sharifi M. Moghbelinejad S. (2018). CRISPR/Cas9, a new approach to successful knockdown of ABCB1/P-glycoprotein and reversal of chemosensitivity in human epithelial ovarian cancer cell line. *Iran J. Basic Med. Sci.* 21 181–187. 10.22038/IJBMS.2017.25145.6230 29456815 PMC5811757

[B17] Peiris J. S. de Jong M. D. Guan Y. (2007). Avian influenza virus (H5N1): a threat to human health. *Clin. Microbiol. Rev.* 20 243–267. 10.1128/CMR.0003736 17428885 PMC1865597

[B18] Reyes-Del Valle J. Chavez-Salinas S. Medina F. Del Angel R. M. (2005). Heat shock protein 90 and heat shock protein 70 are components of dengue virus receptor complex in human cells. *J. Virol.* 79 4557–4567. 10.1128/JVI.79.8.4557-4567.2005 15795242 PMC1069525

[B19] Sato S. Fujita N. Tsuruo T. (2000). Modulation of Akt kinase activity by binding to Hsp90. *Proc. Natl. Acad. Sci; U S A.* 97 10832–10837. 10.1073/pnas.170276797 10995457 PMC27109

[B20] Sauter N. K. Hanson J. E. Glick G. D. Brown J. H. Crowther R. L. Park S. J. (1992). Binding of influenza virus hemagglutinin to analogs of its cell-surface receptor, sialic acid: analysis by proton nuclear magnetic resonance spectroscopy and X-ray crystallography. *Biochemistry* 31 9609–9621. 10.1021/bi00155a013 1327122

[B21] Shin D. M. Yuk J. M. Lee H. M. Lee S. H. Son J. W. Harding C. V. (2010). Mycobacterial lipoprotein activates autophagy via TLR2/1/CD14 and a functional vitamin D receptor signalling. *Cell Microbiol.* 12 1648–1665. 10.1111/j.1462-5822.2010.01497.x 20560977 PMC2970753

[B22] Skehel J. J. Wiley D. C. (2000). Receptor binding and membrane fusion in virus entry: the influenza hemagglutinin. *Annu. Rev. Biochem.* 69 531–569. 10.1146/annurev.biochem.69.1.531 10966468

[B23] Tadasuke N. Fumitaka M. Atsushi K. Kyosuke N. (2007). Involvement of Hsp90 in assembly and nuclear import of influenza virus RNA polymerase subunits. *J. Virol.* 81 1339–1349. 10.1128/jvi.01917-06 17121807 PMC1797515

[B24] Triantafilou K. Triantafilou M. Dedrick R. L. (2001). A CD14-independent LPS receptor cluster. *Nat. Immunol.* 2 338–345. 10.1038/86342 11276205

[B25] Tsutsumi S. Neckers L. (2010). Extracellular heat shock protein 90: A role for a molecular chaperone in cell motility and cancer metastasis. *Cancer Sci.* 98 1536–1539. 10.1111/j.1349-7006.2007.00561.x 17645779 PMC11159077

[B26] Wang G. Jiang L. Wang J. Zhang J. Kong F. Li Q. (2020). The G Protein-Coupled Receptor FFAR2 Promotes Internalization during Influenza A Virus Entry. *J. Virol.* 94 e01707–19. 10.1128/JVI.017071719 31694949 PMC6955252

[B27] Wang R. Zhu Y. Zhao J. Ren C. Li P. Chen H. (2019). Autophagy Promotes Replication of Influenza A Virus In Vitro. *J. Virol.* 93 e01984–18. 10.1128/JVI.01984-18 30541828 PMC6363991

[B28] Wang S. F. Huang J. C. Lee Y. M. Liu S. J. Chan Y. J. Chau Y. P. (2008). DC-SIGN mediates avian H5N1 influenza virus infection in cis and in trans. *Biochem. Biophys. Res. Commun.* 373 561–566. 10.1016/j.bbrc.2008.06.078 18593570 PMC7092884

[B29] Wang Y. Li Y. Ding T. (2017). Heat shock protein 90β in the Vero cell membrane binds Japanese encephalitis virus. *Int. J. Mole. Med.* 40 474–482. 10.3892/ijmm.2017.3041 28656253 PMC5505021

[B30] Wei L. Yong L. Shengxi G. Jianhua F. Chieh-Fang C. Bright A. M. (2014). Extracellular heat shock protein-90alpha: linking hypoxia to skin cell motility and wound healing. *Embo. J.* 30 5022–5022. 10.1038/emboj.2011.420 PMC1817627 17304217

[B31] Xiao X. Wang W. Li Y. Yang D. Li X. Shen C. (2018). HSP90AA1-mediated autophagy promotes drug resistance in osteosarcoma. *J. Exp. Clin. Cancer Res.* 37:201 10.1186/s13046-018-0880886 PMC6114771 30153855

[B32] Xu Y. Jagannath C. Liu X. D. Sharafkhaneh A. Kolodziejska K. E. Eissa N. T. (2007). Toll-like receptor 4 is a sensor for autophagy associated with innate immunity. *Immunity* 27 135–144. 10.1016/j.immuni.2007.05.022 17658277 PMC2680670

[B33] Yang J. Li M. Shen X. Liu S. (2013). Influenza A virus entry inhibitors targeting the hemagglutinin. *Viruses* 5 352–373. 10.3390/v5010352 23340380 PMC3564125

[B34] Yeganeh B. Ghavami S. Rahim M. N. Klonisch T. Halayko A. J. Coombs K. M. (2018). Autophagy activation is required for influenza A virus-induced apoptosis and replication. *Biochim. Et. Biophy. Acta* 1865:S0167488917302896. 10.1016/j.bbamcr.2017.10.014 29108912

[B35] Zhirnov O. P. Klenk H. D. (2013). Influenza A virus proteins NS1 and hemagglutinin along with M2 are involved in stimulation of autophagy in infected cells. *J. Virol.* 87 13107–13114. 10.1128/JVI.021482113 24027311 PMC3838240

[B36] Zhou Z. Jiang X. Liu D. Fan Z. Hu X. Yan J. (2009). Autophagy is involved in influenza A virus replication. *Autophagy* 5 321–328. 10.4161/auto.5.3.7406 19066474

[B37] Zuehlke A. D. Beebe K. Neckers L. Prince T. (2015). Regulation and function of the human HSP90AA1 gene. *Gene* 570 8–16. 10.1016/j.gene.2015.06.018 26071189 PMC4519370

